# Statistics of Grave-Yards in Scotland

**Published:** 1855-12

**Authors:** John Webster

**Affiliations:** Physician to the Scottish Hospital.


### ORIGINAL COMMUNICATIONS.

### STATISTICS OF GRAVE-YARDS IN SCOTLAND.

By John Webster, M.D., F.R.S., and F.R.C.P.,
Physician to the Scottish Hospital.

When making a holiday tour through different counties of Scotland, during last autumn, I carefully observed the ages recorded on the tombstones of various urban and rural churchyards and cemeteries, in order to carry out more fully an inquiry originated by me in the last number of the Journal of Public Health.

I now therefore enter again upon the question; believing that the facts obtained, while they verify the conclusions already enunciated, may induce other observers to pursue this inquiry throughout Great Britain, and to communicate the results of their investigations.

In discussing the question of graveyard statistics, I shall give, for the sake of arrangement, and likewise to facilitate subsequent comparisons, first, the data obtained from monumental and other records in six chief towns, situated south of the river Tay, and comprising the central portion of Scotland; secondly, similar facts procured from six towns north of the Tay, which are situated, comparatively, in equally populous districts; and thirdly, six rural localities, all belonging to the county of Forfar; in order to contrast the ages thus ascertained with parallel mortuary annals belonging to urban populations.

When investigating these varied subjects, it ought always to be kept in remembrance, that the inferences deduced should not be too strictly received as absolutely correct, but simply considered as approximations to general truths, obtained by the method now suggested.

### I. TOWNS SOUTH OF THE RIVER TAY.

1. *Edinburgh*. Throughout the numerous graveyards and cemeteries of the capital of Scotland, containing as it does about 161,000 inhabitants, extreme old age was rarely observed on the monuments erected in these repositories, compared with the aggregate numbers. From seventy-five to eighty appeared by no means uncommon; from that to eighty-five, the instances were numerous; whilst ninety occurred in various examples, and some reached even beyond that period; of which the following statement may be given as a summary:—In the West Kirk burying-ground, many varied from 80 to 90; and 2 females were reported, on their respective gravestones, 97 and 98. In the Grey Friars churchyard, whilst numbers ranged from 80 to 89, 3 were 90; 1 being a male and 2 females. In the Canongate burying-ground, I only observed one monument which reported the deceased person at 90, that of a female; but numbers were recorded from 80 to 88, and under. In the Grange cemetery, two inscriptions, on separate gravestones, stated the individuals—a man and a woman—buried, to have been 93; whilst, by the records of this burying-ground, it appeared that 24 persons from 80 to 89, a male 91, a female 95, and a man 100, had been recently interred. In the Warriston cemetery, from 85 to 89 were frequent; whilst 1 man and 1 woman were stated to have been 90, a man 92, a female 94, and another 98, were instances also recorded; whilst, in the register of this place, 37 individuals are reported to have been interred, from the ages of 80 to 89; 3 men at 90; and 9 from that age to 93—all females, with 2 women at 96; and lastly, 1 woman had actually reached her 100th year. In the Dean cemetery, the ages mentioned on gravestones, being in no way remarkable, need not be specified; although several men were reported, in the register of this place, to have arrived at ages from 90 to 94; whilst 2 women were 93, and one 98, at their decease. Other burying-grounds I likewise inspected; but the different data recorded being much of the same character, it seems superfluous to specify particulars.

Reviewing all the facts collected—and they were numerous—the Scottish metropolis did not seem unusually favourable to longevity; seeing that few residents, in proportion to the large population, attain extreme old age. Very few, comparatively speaking, have lived beyond 85; and many of those who actually exceeded that period were strangers. The opinion now expressed, as to Edinburgh not being very remarkable for longevity, is further proved by the fact that, according to the census of 1851, there were only 22 persons then living in this city, who were 90 years old or upwards, 8 being men and 14 women; whilst only one of these was a centenarian, viz., a female, who had attained 102 years. At present, the oldest resident, according to report, is a female now in her 105th year.

This ascertained rarity of patriarchal persons in the capital of Scotland has been ascribed to various causes; amongst others, to the baneful effects of north and east winds, during winter and spring months, upon persons constitutionally weak in the respiratory organs. To these winds this city is subjected in an eminent degree, from its exposed situation; and these influences are further greatly aggravated by a want of *shelter*, which constitutes frequently a preponderating condition affecting health and longevity in the modern Athens. However, some residents have lived to a very great age: for example, the Canongate bellman, William Edie, who died in 1731, in his 120th year. Of this person report says, he had buried the inhabitants of the district thrice, was ninety years a freeman, and actually married his second wife when one hundred years old.

2. *Glasgow*. Regarding this important populous city—the commercial metropolis of Scotland—with its 380,000 inhabitants, I may premise generally, that the mortuary details collected proved highly instructive; and in reference to infantile life, with its often early termination, were really fearful to contemplate. In short, the data obtained regarding this district assumed altogether such a character, as must arrest the attention of all sanitary investigators, equally with philanthropists. In the old burying ground near the Cathedral, as also in the new Necropolis adjoining, very new grave stones indicated persons there interred to have been beyond 80; although the registers showed that several from that age up to 85 and 88 had been buried; whilst a gravedigger mentioned that some years ago a man upwards of 100 was deposited near the cathedral.

In the Sighthill Cemetery—the most extensive burying-ground within this city—80 to 85 were very unusual recorded ages, either on the grave-stones or register, but 30 to 50 appeared exceedingly numerous: but it was stated, that an individual aged 103 had been buried in this locality some time previously. In the southern Necropolis—also a large burying-ground—several persons interred had reached the age of 80 to 85; one was 88, a female 91, another female 93, and one man had attained 98 years at death; still the great majority were much younger. In the Gorbals old churchyard, I saw no age stated upon any grave-stone beyond 84; one was 83, and several from that period down to 80, many however being far below these figures. Other public receptacles for the dead, of which there are upwards of twenty, might be specified; but the general observations are of much the same character as those already detailed.

Maturely weighing all the facts obtained, whether from inscriptions on grave-stones, from the registers of burials in cemeteries, many of which I carefully examined, or from the valuable official sanitary reports, which have been for some years past regularly published, to illustrate the social and economic statistics of Glasgow, it may be fairly asserted, that this locality is neither salubrious nor remarkable for the longevity of its inhabitants, when compared with numerous other populous districts. Many born within the city and suburbs, die in early life; 44·50 per cent. of the population perish before they attain the age of five years; whilst the still-born amount to about one thousand annually. The deaths during adult life are also very large in proportion, since 84 in every 100 persons born never pass their 60th year; and further, among the 93,543 registered burials, during the last seven years, only thirteen persons were reported as centenarians. Collaterally with these facts, and as corroborative evidence, it may be mentioned that in 1851, amongst the whole population of Glasgow, estimated at 329,097, only 79 individuals were 90 years or upwards, and not more than four above 100 were stated to be then living. An authority of some weight, who spoke from his own knowledge, stated to me in conversation, that few persons, comparatively speaking, who have been long residents in this city, ever pass their 80th year; whilst among those reported to have died beyond that period, a large proportion were persons from the country, who had come to reside with relatives, and thus benefit by their bounty and protection.

Numerous causes have been assigned to account for the great mortality of Glasgow, and the inferior position it occupies in the longevity scale. Its damp and generally low situation; the insalubrious and often dangerous employments in which many persons are engaged; the unhealthy atmosphere, polluted by thick smoke or noxious vapours from chemical works and furnaces; the mixed and varied races who dwell in crowded courts; the ill-ventilated dwellings; the spare living; the vegetable diet; whiskey—Scotia's bane; exposure to a moist climate; cold weather; imperfect clothing, which prevails especially amongst women and children of the lower ranks, whose necks, extremities, or heads have very seldom any covering, and who frequently walk the streets, whatever the season, without either shoes or stockings—all these influences, irrespective of others, which are constantly in operation throughout very large and crowded communities, have contributed to affect so deleteriously the public health of Glasgow. But, whatever may actually have proved the chief causes, there cannot exist any doubt respecting the conclusion already enunciated, viz., that this city is neither healthy as a residence, nor remarkable for the longevity of its inhabitants. There is, however, now living in *the neighbourhood*, a lady in her 107th year, and very recently another female was reported to have died at the age of 101: whilst a carpenter named John Walney died in Glasgow during 1757, actually 124 years old. This man is stated to have married eleven wives, all of whom he buried; and of his seventeen children five survived him, whose ages amounted to 326 years collectively.

3. *Greenock*. In the graveyard and cemetery of this beautifully situated town, with its numerous splendid aquatic mountain prospects, the ages recorded on several monuments spoke rather favourably as to its general salubrity, and the longevity of its inhabitants. The actual position occupied in the mortuary scale by this town, with at least 38,000 residents, although not very high, seemed satisfactory. From 80 to 85 appeared not unfrequent ages, considering the population. Some recorded ages were 87; but the highest I observed on any monument was 90, although a few older persons than the above lie interred in the new cemetery. Recently, the mortality has been considerable at Greenock, especially amongst young persons; more than half the aggregate deaths having occurred in children under five years. Notwithstanding these facts, this district may be reckoned superior in salubrity to Glasgow, but under several towns to be noticed subsequently.

4. *Stirling*. This ancient city, having near 13,000 inhabitants, and celebrated for its castle, and for its splendid prospect over the fertile vale of Forth, gave good grave-yard evidence as to the position of this district in the scale of longevity. Many persons interred in the parish churchyard and in another burying ground were from 75 to 80, some reached 83 and beyond; one was 86, another 89; whilst the oldest person recently buried was the late grave-digger, who died at the advanced age of 94, after having filled office upwards of thirty years. Although not the oldest age observed, nevertheless, one gravestone may be here noted, that of a patriarchal member of the medical profession, Dr. Abraham Gordon, formerly resident in Stirling, who died at the age of 86. From the different data obtained, Stirling exhibits a medium longevity, and on the whole may be considered as salubrious, notwithstanding the exposed position of the castle and lower portion of the town, which lies near the river Forth. The declivity of the principal street, which descends from the castle, affords a constant fall for the rain currents, and must be beneficial, in carrying away every kind of filth more readily.

5. *Perth.* "The fair city," as it is often called, the population of which is near 26,000, next comes under review. The situation it occupies is a meadow close to the river, having the beautiful hill of Kinoull on one side, and the equally famed hill of Moncreiff on the other, whence the Roman soldiers of old, when approaching Perth, and first beholding the Tay, are said to have exclaimed, "Ecce Tibur," so magnificent seemed the scene around.

Notwithstanding several local causes inimical to health and longevity, Perth appeared, after careful inspection, rather better than I at first expected. The ages recorded on gravestones, observed in its burying-grounds, were in many instances from 70 to 80; numbers reported the ages of 81 to 85; some were 90, and one was 93, a female. Besides these monumental statements, it is important to add that, in the corporation records which I examined, it was stated that twenty-five persons whose ages exceeded 95, had been interred in the city burying-grounds, during the last nine years, besides seven centenarians, who had been likewise buried during the same period: and at present there is still living in Perth a lady in her 100th year. These data are important in reference to the question in hand, and certainly constitute very conclusive evidence regarding the longevity which the residents of this city have in many instances attained.

6. *St. Andrew's.* This classic city and ancient metropolitan see of catholic Scotland, at present having about 5,200 inhabitants, takes a high position in the longevity scale. In the large burying ground of this recently much improved place, surrounding the ruins of the formerly magnificent cathedral, ruthlessly destroyed in 1559 by the reformers, numerous grave-stones show that advanced ages are common in the locality. Many persons interred were from 70 to 80; numbers exceeded that term to 85, several reached 86, others 87, 88, and 89, and even 90; whilst six individuals reached from the latter age to 94. In reference to the above patriarchal persons, it is a curious circumstance that, amongst the very oldest, a great majority were men; whereas the females who attained the highest age observed on any monuments were 87, of which however three examples were noticed. Of the former sex one memorial merits an especial notice; namely, that of Professor Ferguson, the celebrated philosopher, and previously travelling companion to Lord Chesterfield of courtly fame. This Nestor of intellectual men lived many years in St. Andrew's, and died aged 93, according to the inscription on his monument in this cemetery.

Besides the data now reported, I may add, on the authority of Dr. Adamson, a physician of repute in St. Andrew's, who has paid much attention to sanitary questions, that out of 311 deaths reported by him on a recent occasion, when illustrating a point he was then investigating, 83 were persons above 70 years old; 41 beyond 80; 11 more than 85; and 2 over 90 when they died; all of these being inhabitants of the neighbourhood specified.

Restricting any conclusions regarding the longevity of persons in this part of Scotland to the ascertained facts now detailed, it may be correctly asserted that, the city of St. Andrew's is a salubrious residence, whilst its residents are distinguished for the advanced age which they often attain. Nay, if all gravestone stories be authentic, this part of Fifeshire may boast of possessing the bone of one of the most venerable patriarchs amongst the human race, recently seen on earth; he being even older than Abraham, who died at the great age of "one hundred, three score and fifteen years," or Jonas, who was 180 years old. The modern Methuselah now alluded to was named Watt, and by occupation a gardener; he died in 1800, according to a statement on the large horizontal stone slab placed over his grave, near the cathedral ruins; which record states that Watt was "one hundred and eighty-five years at death"! The figures announcing this extrordinary circumstance are cut deeply into the stone, apparently all at the same time, and coeval with the inscription, detailing the name, occupation and so forth, of the party in question. However, after minute examination, it struck me that the figure 1 had possibly been interpolated. At all events, I now give this explanation, with my scepticism respecting the age inscribed. Subsequently I stated the above gravestone legend, and this opinion, to several university dignitaries, learned professors, and others; but none had heard of the monument mentioned. Doubtless, future investigation will be instituted, to ascertain the exact history of Watt; and if he really did attain 185 years, so rare a phenomenon ought to be authenticated by unquestionable evidence.

### II.—TOWNS NORTH OF THE RIVER TAY.

1. *Dundee.* The first town comprised in this division is an important commercial maritime port, upon the north bank of the river above named. It is about ten miles distant from the German ocean, and occupies a declivity, gradually rising towards the hill or "Law" as it is often called, which reaches to upwards of 550 feet in height. This elevation, designated in Celtic "dun", and giving origin to the name, Dun-tay, but now corrupted into

Dee, although called "Taodunum" by the Romans, shelters the place from northerly winds, and so moderates its temperature. The population is about 80,000, of whom many are employed in linen and yarn factories,—the staple manufacture,—in ship building, iron founding, and other dangerous occupations. Hence, although favourably situated in some respects, with a southern exposure, and possessing other satisfactory features, this locality is considered to a certain degree unhealthy; particularly through exhalations arising from "silt" on its shores near the harbour, which is uncovered twice a day by the tide receding.

In the old burying-ground, or "Howff", situated in the centre of Dundee, a large proportionate number of gravestones indicated 70 to 80 as very common ages amongst persons here interred; many exceeded 80 to 85; and from that to 90 was not uncommon. Beyond the latter period to 95, several instances were observed; the highest ages I recognised being 97, of which two examples occurred, one in a man, the other in a woman. In the new burying-ground, in the Western cemetery, and in minor graveyards, the recorded ages being much of a similar character, did not materially alter this general inference, arrived at after examining various data, viz., that Dundee holds a high position in the longevity scale, especially when compared with many other sea-port and manufacturing populations.

Having been favoured, by the kindness of Mr. Baxter, town chamberlain, with official returns of all the burials in this town and suburbs, during the last twenty-one years, I would also direct attention to an analysis of these valuable reports, compiled after some labour; since several very interesting conclusions may be deduced with considerable confidence, which amply illustrate the vital statistics of this part of Scotland.

Throughout the period above specified—twenty-one years ending 31st December, 1854—the total interments within the district amounted to 37,500 of all ages, amongst whom nearly one-sixth, or 17·45 per cent. were infants and children not exceeding one year old. From that period to 5 years, the proportion of burials was 19·60 per cent. Up to 20, the ratio became nearly 10 per cent.; hence showing that, about 47 per cent. of the aggregate burials were persons under their twentieth year. In other words, almost half the deaths recorded in Dundee occurred amongst individuals who had not arrived at the period of manhood. From that epoch to 50 years, the deaths reported reached nearly 22 per cent Up to 70, the ratio was 14 per cent. To 80, it ranged 16·16 per cent.; and as 2310 persons died from their 70th to their 80th year, it appears that a large proportion of residents really attained an advanced age. Between 80 to 90, the total deaths amounted to 814; which indicates that 1 person in every 46 interments recorded, lived beyond the eightieth birthday, and some much longer. From the last named period to 100 years, as many as 94 cases were enumerated; from which it follows, that 1 person out of every 400 burials lived until the very unusual period just designated. Of centenarians, 8 instances were recorded during the entire 21 years; amongst whom females were most numerous, being in the proportion of five to three. Only 1 person exceeded 102 years; a man who attained the patriarchal age of 114 years, and was then considered the Nestor of Scotland, although not a Scotchman by birth. He was born at Bath, in England.

Since the place of nativity is generally reckoned a question of interest, if not of great importance, in reference to longevity; and as some districts often claim the prestige of being the birthplace of more persons who live long, than other localities less favoured in that respect, it becomes instructive to ascertain, whether many of the individuals who passed 90 and beyond 100, were natives of Dundee. Upon analysing the tables already quoted, it appears that, amongst the 111 residents occupying the venerable position of 90 years of age and upwards, 18, or one-sixth of the whole, were born in this district; but only one, a female, was classed as a centenarian. I would also remark, in reference to the sex of very old people, that amongst the above 111 persons, 36 were men and 75 women, *i.e.*, two of the latter to one of the former. Further, it should be specified, that 8 of the 111 were centenarians; of whom, besides the female born in Dundee, one was a native of Brechin, and another of St. Vigians, thus making three persons who were natives of Angusshire; whilst another was born in Ross-shire, the fifth in Inverness-shire, the sixth near Blair in Athol, the seventh in the Isle of Skye, and lastly, the eighth was the Englishman, born at Bath, as already mentioned. From such data, it may be deduced that females have twice the chance of becoming nonagenarians, compared with males.

In concluding these observations on the vital statistics of Dundee, I would draw attention to the large number of still-born children annually reported within its precincts: 2701 cases having been so recorded, during the 21 years already quoted; affording a ratio of 7 and nearly one-fifth per cent. of examples of this class, a proportion even larger than in Glasgow: seeing that the exact number of still-born children in that city was 6619, out of 93,543 burials. Statements like the above, based upon official documents, are remarkable, and demand serious attention by the legislature.

Compared with other large towns, or even capitals, both in England and on the continent, the amount of still-born children does not often attain to so high a figure comparatively, as in several districts of Scotland. For instance, in London the ratio is about $3\frac{3}{4}$ per cent. of the total deaths registered. In Berlin it falls under 5 per cent. At Stockholm the proportion is less than 4 per hundred. In various large towns of France, the number of still-born children is below either Glasgow, Dundee or Arbroath. Thus, in Bordeaux it ranges under $2\frac{1}{2}$ per cent.; in Rouen less than $5\frac{3}{4}$; in Lyons $6\frac{3}{4}$; in Paris $7\frac{1}{3}$ per cent., or nearly the same ratio as in Dundee; but in Marseilles it rises higher, reaching to

$7\frac{3}{4}$ per cent. These varied statements are curious, and have now been quoted to illustrate the important question brought under discussion.

*Arbroath.*—This manufacturing town lies on the sea-coast, near the embouchure of the river Tay. The place is densely peopled for its size, having about 17,000 inhabitants; and as the staple manufacture consists chiefly of flax, many residents follow occupations often not very salubrious. Certain peculiar features characterising this locality merit special mention; first, the number of tall, huge, smoking chimneys, belonging to factories—upwards of thirty—which are crowded together almost like a forest, within a very limited space; and secondly, the singular phenomenon of a river of boiling water, the Brothock, running into the sea, while giving out volumes of steam, only a moment before condensed by the engines at work in various establishments. I have never before witnessed such an occurrence, and the fact is now mentioned chiefly on account of its great rarity, rather than from any known effect produced upon public health.

In the old and very crowded burying-ground, near the magnificent remains of the ancient abbey, destroyed, like St. Andrew's, at the Reformation, many gravestones shew that the ages of parties interred were from 70 to 80; numbers from that age to 85; and not a few from 85 to 90; whilst several ranged betwixt that period and 94, which were the highest figures seen on any monument. The majority of these very old people were females. One man, however, should be mentioned; namely, a late gravedigger in this cemetery, who lived till he attained 93 years, having, like the same official dignitary at Stirling, followed his calling during the third part of a century. These data, besides the fact that there is now living, near this locality, a female in her 94th year, whom I know personally, shew that Arbroath stands well in reference to longevity; and that, if not on a par with St. Andrew's, it is far superior to several other manufacturing maritime towns, especially Glasgow.

The reference just made to the great age of the respective grave-diggers at Stirling and Arbroath, notwithstanding the admitted insalubrity of their occupations, deserves notice; especially as other instances of a somewhat similar kind have been remarked elsewhere. Of this fact I will, however, only now mention one instance which came to my knowledge, viz., that of a man named Barton, who had acted as sexton at Horsely, in Derbyshire, during seventy years; whilst his wife practised as a midwife for the long space of eighty years, and died at the age of 107. Of these rural functionaries, it is said, that *she* had twice brought into the world, *he* had twice buried, the whole parish. Coupling the above-cited cases with that of Watt, the St. Andrew's gardener, Shakespeare's well known saying, uttered by Hamlet, "There is no ancient gentlemen but gardeners, ditchers and grave-makers," seems completely verified.

Anxious to procure additional facts illustrative of longevity, I

obtained, through the kindness of my friend, Mr. James Anderson, an account of all the burials in this district during the last ten years; from which it appears that, out of 3112 interments, 164 were of persons aged from 80 to 90 years; 19 were above that period and up to 95; 4 were 96, and 3 had attained their 97th year; but none were older; consequently, centenarians appeared very rare in Arbroath during the period embraced by this official document; indeed, no one beyond the venerable age of 97 had been recently buried. In reference to the general mortality, it follows, from an analysis of the table just quoted, that more female than male persons were interred; the excess being nearly 5 per cent. From 20 years of age to 50, or the period of manhood, there died upwards of one-fifth, or 637 persons out of the total of 3112; from that period to 70, when physical strength begins to fail, a sixth, or 528; whilst, betwixt 70 and 80, at which period most men or women become really old, nearly one-tenth, or 305 persons, passed to their long home. Lastly, infants and children under 1 year ranged rather below one-sixth of the aggregate burials, viz., 526 cases; which constitutes an important fact regarding infantile life, and shews that it is even more precarious in Arbroath than some other manufacturing towns; as for example, Glasgow, where, notwithstanding its admitted unhealthiness, and other causes inimical to the health of very young persons, the proportion of deaths amongst individuals of that category was 14·28 per cent.; whereas, in the town of Arbroath, the ratio reached 16·87 per cent., or upwards of 2·5 increase. This result is important, and shews that some injurious influence in operation demands special attention.

3. *Forfar*. Forfar is an inland county town having about 9300 inhabitants, and resembles Arbroath in reference to the chief occupation of its labouring population, the staple employment being linen manufacture. It differs, however, from that place, in not possessing many smoking factory chimneys to pollute the atmosphere, and in not being a seaport. Nevertheless, from its vicinity to a loch, formerly more marshy than now, and having rather elevated ground towards the south, which diminishes to a certain extent the influence of sunshine, and from being likewise rather exposed to east or northerly winds, this district labours under several local disadvantages in respect to salubrity.

In the old churchyard, now almost disused for interments, and in the really beautifully arranged new cemetery, from whence an extensive yet splendid view of the Grampian range is obtained, many persons of advanced age have been buried. Numbers were from 70 to 80, many from that age to 90, and several even up to 95; and the oldest person, a man, recently deposited in the cemetery had attained 99, although no gravestone inscription yet records the death of that all but centenarian individual. Besides these examples of old age, in the episcopal chapel a tablet records the age of a widow lady connected with a branch of my own family, who died recently at the age of 96, her place of birth being in the neighbourhood. Another illustration of great longevity in this district is that of an old man I knew, who lived within a few miles of Forfar, until he had attained his 105th year.

Access having been given me, through the kindness of a friend, to the town register of burials kept during recent years, it will prove a curious and useful task to state the general results thereby procured respecting the ages of persons interred within the borough precincts, and to illustrate the longevity of residents.

According to this official register, embracing about 2,000 burials of all ages, it appears that 64 persons had completed their 80th year at death; of whom 43 ranged from the above term to 85; 16 exceeded the latter period to 90, three being in that category; whilst one died at 91, two at 92, and one at 95, 96, 98, and 99 respectively.

Reasoning from all the facts above detailed, Forfar does not seem very remarkable in reference to extreme old age; for, although standing well in the longevity scale, it occupies a lower place than some districts previously described. Its oldest resident, at present, is a relative and namesake of my own, who stated, during a visit I paid her, that "very soon she would be nearer 100 than 90 years." Her faculties were still good, and her bodily health appeared satisfactory; in proof of which, one remark made to an acquaintance after this interview may be quoted, viz., "Dr. Webster is very like his grandfather Bailie John," so designated in 1750, when he became a civic magistrate, and whom she remembered perfectly, notwithstanding that he was born early in the last century. It is likewise interesting to add to the above fact, that there lives with this venerable gentlewoman a younger sister, now in her 93rd year, the junior scion of such a long lived race, enjoying also equally good, if not better health than her senior in years. Both are natives of Forfar, and have lived long on the north side of a street lying east and west, exposed to westerly winds, and to sunshine; influences—especially the latter—always conducive to health and longevity.

4. *Brechin*. The city now mentioned is situated in an inland district, about eight miles distant from the German ocean, and contains about 6,650 inhabitants. It occupies a declivity upon the northern bank of the south Esk, but several streets and alleys chiefly occupied by its manufacturing population being placed on low ground, adjoining that river, are hence less salubrious; and whenever any epidemic disease prevails, it rages usually in this quarter. Not being a manufacturing town to any extent, although there are still in this locality numerous ill paid hand-loom weavers, Brechin must not be placed in the same category as either Dundee or Arbroath in reference to its population, or their employments, with the excitement consequent upon great mercantile pursuits. Besides, it is not a seaport, and therefore remains free from the physical evils and contaminations, as well as moral degradation, so commonly characteristic of such places. Altogether, this district, from the absence of several influences inimical to the health of residents, stands forth favourably in regard to longevity.

Within the now over-crowded churchyard adjoining the celebrated Pictish round tower, one of the greatest curiosities throughout Great Britain, and which has given rise to much learned controversy, the gravestones show that many persons of advanced life have been interred. In this romantic looking precinct, ages betwixt 70 and 80 are frequently recorded. From 80 to 85 were not uncommon, considering the aggregate monuments. Several ranged from 85 to 87, which were the highest figures observed. In addition however to the above statements, it deserves to be mentioned that, although no monumental stones yet record the various facts, five persons from 82 to 85, one 86, another 87, one 88, one 89, and lastly one 93 years of age respectively, have been recently interred in this ancient cemetery. Besides the above facts, there is now living a female aged 96, and another not much younger, who are said to be the oldest living persons at present in Brechin. According to these different data, the inhabitants of this city seem therefore to enjoy above an average, if not rather a high position in the scale of longevity; fewer persons apparently being cut off during middle life than occurs in crowded manufacturing localities, especially those lying on the sea-coast.

5. *Montrose.* This town is a seaport with about 16,000 inhabitants; it occupies a neck of land, chiefly of sand. It has the ocean on one side, a tidal river on the other, an equally large stream on the northern quarter, but at some distance; and lastly, an extensive natural basin or lagune of several square miles in extent on its western boundary. The town is not very favourably placed as to salubrity: for the muddy bottom of this large marine lake being exposed to the action of winds and sunshine, damp exhalations, with other injurious malarious influences, are liable to arise, and affect visitants. To make matters much worse, an extensive manure manufacturing establishment has been lately, by magisterial sanction, allowed to occupy a ground lying between this lagune and the high street of Montrose; from this manufactory most offensive stenches often exhale, which are felt over great part of the town, especially when westerly winds prevail. I can speak on this point from personal experience; for, on the day of my visit to the lunatic asylum and graveyards, I passed this manure depôt, at the time a west-wind blew from over the mud encumbered marshy basin, then uncovered by sea water, and exposed to a burning sun's rays. The odour produced was so strongly unpleasant, that, like Faraday going down to Greenwich by the steamer, I was constrained to put a handkerchief to my nose, to avoid smelling the putrescent effluvia. This is really no exaggeration, for others have made similar remarks respecting this stercoraceous magazine; and the subject is here mentioned, since the evil might be easily remedied, if only to the public good the paltry annual rent received from lessees was sacrificed. To talk of promoting sanitary measures on one hand, and to allow these noxious proceedings on the other, is altogether inconsistent.

Like Dundee, Arbroath, and Brechin, the old burying-ground of Montrose is situated in the centre of the town, close to its principal street, and, being much crowded, is most objectionable, besides being injurious to public health. However, interments have for some time past been very properly discontinued, and burials now only take place in other cemeteries. Many of the gravestones in these localities indicated the ages of persons interred as varying from 70 to 80, although numbers on the other hand were much younger. From 80 to 85 appeared not infrequent. Several in the old churchyard ranged between the latter age and 90; but of this only one monument was observed. It deserves special mention. This monument had been erected by a venerable family named Booth, to commemorate the father who died at 90, and a son at 84; the mother being 89 at death. Its inscription stated that one of the former had acted as superintendent of the Montrose lunatic asylum upwards of 40 years; so that, in many respects, this mortuary memento deserves notice by modern "Mortalitys" searching for examples of long lives in the same households.

Judging from the facts specified, Montrose is not entitled to a very high position in the longevity scale, nor does it occupy an inferior place amongst manufacturing and marine populations, both of which features, speaking generally, this town exhibits. Nevertheless, some very old people have been met with amongst residents, as for instance, the venerable mother of a friend of mine, and an M.P., who lately died here at the age of 96. On the north and south elevated ground which rises upwards from the two rivers Esk, examples of long-lived people have rather frequently occurred; a recent illustration being afforded in a gentleman who many years resided on the southern elevation, who had entered his 97th year at death. The immediate vicinity of Montrose may therefore be considered salubrious.

6. *Aberdeen.* The university city of Aberdeen contains about 74,000 inhabitants. This granite northern metropolis, as it is often designated, occupies a situation moderately elevated, but sloping in some places towards the river Dee, where large docks have been constructed. Like Montrose, the ocean washes on the eastern side, whilst another river, the Don, bounds old Aberdeen on the north; but no tidal lagune here sends forth, from its western boundary, malarious exhalations. Many of the ancient streets are narrow, with confined tortuous alleys and courts adjoining; but the chief thoroughfares and squares are wide, and possess free ventilation. Being both a seaport and manufacturing district, several influences inimical to salubrity prevail in this locality; still, as the city has a dry granitic foundation, an abundant supply of excellent water, with other local advantages, its climate, in spite of frequent cold and stormy weather, may be reckoned on the whole as healthy, unless for very young people, and those possessing feeble corporeal frames.

When perambulating Aberdeen, especially during one Sunday, the weather being fine and enlivened by sunshine, I was enabled to observe more extensively its teeming population. I was then much struck by the tall figures, strong physical conformation, and outward favourable aspects of the numerous persons whom chance thus brought under observation. Similar to the East Angles, alluded to in my former communication, the Aberdonians likewise supply many excellent specimens of the "genus homo"; whilst their reputed quick intellect, and often high mental endowments, fostered and improved by the diffusion of good general education amongst the masses, from having two universities, with other advantages, place natives of this classico-mercantile region of Scotland in a favourable position amongst their countrymen. Were whiskey-drinking less frequently indulged in by the lower classes; if no seaport existed, always causing certain evils amongst its variable and often reckless population; and lastly, but not the least importantly, were residents always fully employed and amply remunerated for their labour, so as to enable them to obtain abundant necessaries of life, with more creature comforts than many have of late been able to procure, this northern district would, doubtless, have become even more remarkable in many respects than it now is, as regards salubrity. Longevity is one of the characteristics of this part of Scotland. This may be shown, irrespectively of any data detailed in subsequent paragraphs, by the well known fact that many speculators on the London Stock Exchange have often selected aged inhabitants living in this neighbourhood as lives upon which to purchase annuities; by this process considerable sums of money were once gained at the expense of government, and a former chancellor of the exchequer was not a little mystified. At length some check was put upon these gambling proceedings, heavy losses having been entailed on the public revenue from the unexpected vitality of numerous annuitants resident in this northern district.

In the old burying-ground adjoining Union Street, the very centre and most frequented part of Aberdeen, as also several new cemeteries in its vicinity, numerous gravestones record a number of persons interred therein at ages from 70 to 80; whilst a larger proportion than ordinary ranged from that period to 85; numbers were from 85 to 90; and beyond that limit, up to 95, were many more instances than I have anywhere else observed; the last quoted figures marking the highest recorded age which came under my observation. In old Aberdeen churchyard, however, several tombstones bore witness of even more advanced ages. Besides many from 70 to 80, a large proportion ranged from that to 85; numbers were even as high as 90; that term, as also 95, supplying various examples. One monument had 97 as the age of the person interred, being that of a man; whilst another male named "May" was stated to have lived to 102 years; the highest churchyard recorded age met with during my peregrination through Scotland.

In the Foot-Dee churchyard, chiefly used as a burying-place by residents in the low lying portions of Aberdeen, near the harbour, the examples of advanced ages registered on gravestones were scarcely so numerous in proportion to the aggregate number as those met with elsewhere. Nevertheless, besides many about 70, some were from 80 to 85 and upwards; several ranged from 85 to 90 inclusive; one person, a male, was 92; and two persons, a man and woman, were reported as 96 at death. This locality cannot be considered so favourable to longevity as the more elevated situations. Irrespectively, however, of this exceptional inference, the comparatively greater span of human life often enjoyed by inhabitants of Aberdeen is indubitable; and this opinion is farther supported by the fact that, during the first six months of 1855, when 907 deaths were registered throughout the two registration districts of the city and suburbs; not fewer than 54 persons died betwixt 80 and 85, while from the latter age to 90 there were 13 instances; up to 95 also 5 examples were reported; whilst two died at 97, one at 98, another at 99; and lastly, a female recently buried was stated to be 108. This centenarian had resided close to Aberdeen for many years, and is included in the register of Old Machar.

Notwithstanding the admitted fact that many persons attain a very advanced age in this part of Scotland, it is equally true that the climate of Aberdeen is not favourable to juvenile life; hence the proportion of infants and children, cut off ere they arrive at full growth, is considerable. In illustration of this feature I may mention that, on analysing the 568 deaths recorded during the first half year of 1855, in the registration district of St. Nicholas, which includes the old part of New Aberdeen, 112 cases, or nearly 20 per cent. of the whole, died in the first year; from that period to 5 years inclusive, 44 died, or 7·74 per cent.; after that age to 15, also inclusive, 42 died, or 7·39 per cent.; from 15 to 25, the deaths were 43, or 7·57 per cent.; after the latter age to 58, the term of full manhood, when mental and bodily vigour become often taxed to the utmost, 131 persons fell victims to disease, thus making 23 per cent. of the entire number. From thence to 70, by which time the human frame begins to get old, even under favourable circumstances, 79 individuals, or nearly 14 per cent. were called to their final home. Beyond that epoch, and up to 80 years of age 68 persons, or almost 12 per cent. ceased to exist. From 80 to 85, when all are really old, 23 died, or 4·04 per cent. After that age to 90, the deaths were nine; and lastly only two exceeded the last named period, one being 93, the other 97 at death. These different authentic data would therefore seem to demonstrate, in reference to the question of longevity, that, prior to the human constitution attaining maturity, or 25 years, in this district of Aberdeen, not the most salubrious, upwards of four-tenths of all persons born succumbed to disease; whereas, 102 individuals, being nearly one-fifth of the whole, lived till they had completed their 70th year, and even much longer, as stated in a former paragraph.

Before taking leave of this part of Scotland, and its gravestone records, I would add by way of conclusion, that many of the very advanced ages occurred in the city clergy, and the professors of both colleges; a circumstance which is by no means general. Besides this peculiarity the proportion of old men appeared to preponderate over women, a feature rather uncommon in other districts.

The longevity of the Aberdonians is not an event of the present period. A soldier named Alexander McCulloch, who served under Cromwell, and in the three following reigns, died near the city in 1757, at the age of 132 years. Donald Cameron of Kinnichlabar, in Rannach, who married when one hundred, died in 1759, aged 130 years. Another person of the same clan, viz., Archibald Cameron, a piper to seven Lords of the Isles, during the long period of ninety-four years, died at Keith in 1791, aged 122 years. A woman named Catherine Brebner died near Aberdeen in 1762, at the age of 124; whilst another female named Mary Cameron, but whether any relation of the two Camerons above reported does not appear, died at Braemar in 1784, aged 129 years.

### III. COUNTRY OR RURAL DISTRICTS.

The churchyards of these districts are all situated in Angusshire, an agricultural and also manufacturing county. The towns, with one partial exception, are inhabited by persons chiefly engaged in agricultural occupations; they constitute therefore good examples of a rural population.

1. *Tealing.* This rural parish lies about five miles north of Dundee, in a kind of valley south of and protected by the Sidlaw hills on the river Fithie. Its population is about 850 persons; hence the gravestones are not numerous in the burying-ground, and seldom more than fifteen interments take place annually. Several monuments stated from 70 to 80 as the ages of parties buried; beyond that period to 85, there were four instances; betwixt the latter period and 90, I observed three examples; whilst the highest age recorded was 92, being that of a male resident. From these data Tealing occupies a rather high position in the scale of longevity.

2. *Inverarity.* This parish, four miles south of the county town, Forfar, has a population of about 950 inhabitants. It is an entirely agricultural district, on the banks of the small river Kerbet; and as it has no works causing a crowded population, the inhabitants neither live in impure air, nor follow unhealthy employments. Agriculture on an improved and systematic plan is the occupation of the whole neighbourhood, leading to out-door labour, tranquil industry, and regular habits. Further, the authorities of this district have for years past got rid of public houses, with their many attendant evils; this has acted most beneficially. A parish library provides healthy enjoyment for such as seek for mental improvement. The gravestones in the rural churchyard of this parish, not being numerous, supply scanty evidence in reference to the ages of those interred. Nevertheless, I can state on excellent local authority, as well as on personal knowledge, this being my own birthplace and early residence, that Inverarity is salubrious, and that the residents are often long-lived. Thus, out of 117 persons belonging to this locality, and buried during the last ten years in its cemetery, 43 were above 75 years old, and 21 beyond 80 at death; three were upwards of 94, and two had reached 96 when they died, one being a female whom I knew individually. Lastly, there are now living in this parish ten persons who have passed their 80th year. Reasoning upon the above authentic statements, this district is therefore healthy, and characterised by the longevity of its inhabitants.

3. *Aberlemno.* This place is a parish about six miles north-east of Forfar, and celebrated for several curious runic monuments, The population is about 1,100 persons, and the burials average from 17 to 18 annually. In its churchyard various gravestones, especially of male persons, stated their ages to have been from 80 to 85, although several were under; one was 87, whilst the highest observed had reached 92 at death. The oldest resident in this neighbourhood is a female aged 92, named Mary Webster. Aberlemno occupies a favourable position in the longevity scale.

4. *St. Vigians.* This suburban, and in point of extent chiefly rural parish, lies close to the town of Arbroath; indeed it includes a portion of that borough, although recently this part has been divided "quoad sacra." The district extends north-east from the town; and, being so near the sea, its atmosphere partakes considerably of the qualities usually observed in marine residences. In the romantically situated churchyard of St. Vigians, which occupies a small mount, seen from some distance, various gravestones show the ages of 70 to 80 as common. Several ranged from 80 to 85, others from that to 87; but the oldest person buried here was the late clergyman of the parish, a grand-uncle of mine, who lived till he was 93, and now reposes under the identical pulpit from whence he preached during a very long period. It is important to mention that recently three persons died in this district at the age of 90, besides another, a female, who was 103 at her death, and had scarcely ever lived out of the parish. In addition to these facts, it is worth stating that a rather celebrated man in his own day, viz., "Petrus Young," who was assistant to the learned George Buchanan, tutor to King James VI., lies buried in the north vault. This person, sometimes called "King Jamie's pedagogue," died at the age of 83, as mentioned in Latin on an ancient marble tablet placed upon the inner wall of the church.

5. *Lochlee.* The locality of Lochlee is situated among the Grampian Hills, within an entirely highland district. Its population does not exceed 600 inhabitants, amongst whom the deaths generally average from 10 to 12 annually; while, according to recent statements, 25 persons were then living who had passed the 70th year of their age. Here, several persons have died, numerous certainly in proportion to the total population, betwixt 70 and 80. Of deaths at from 80 to 85 inclusive, six instances have occurred within a few years. Two also died at 86, one at 88, and one at 90; the last four persons were all females.

6. *Edzell.* Although partly a highland district, occupying the southern declivity of the Grampian hills, which border the northern limits of Angusshire, this parish is chiefly lowland. Its population is about 1,080, and the deaths average from 18 to 20 annually. In the sequestered burying-ground of this locality, during several years past, a large proportion of the burials included persons who had lived to 70 or 80 years; there were three instances after that period to 85, and four had lived from 85 to 87; at 90 two were recorded, at 91 one, and the oldest person recently buried was 92 at death, the last two persons were both females. At the time of my visit to this burying-ground, where the family vault of the formerly powerful and warlike Lindsays — ancient Lords of Edzell — still exists, an old pauper woman aged 90 was being interred. Taking all the facts I could collect into consideration, this locality stands high in reference to long life. The chief village of this parish, Slateford, is often recommended by neighbouring medical practitioners as a residence for invalids. Winter is often severe, and the adjoining high Grampian hills are sometimes long clothed with their snowy covering; still, as this district lies on the southern side of a mountain range, and is not malarious, it possesses many local advantages which conduce to long life.

### GENERAL REMARKS.

Although numerous interesting deductions might be fairly made from the data contained in previous pages, I shall only add a few general observations, my chief object being rather to bring forward some curious statistical information personally collected, respecting graveyards in Scotland, than to enunciate mere opinions.

According to the statements previously detailed, it appears that old people were more numerous in town districts than most persons would have supposed. Unquestionably, many of the oldest persons recorded on gravestones died in populous towns; but a large proportion of these individuals had migrated from the country in early or in middle life, some towards the evening of their days. Glasgow is an instructive example of this; since, in this city, as I was informed on very good authority, several patriarchal individuals, buried in its cemeteries, were strangers. That towns are inimical to infantile life, there cannot exist any question; seeing that the records of all densely populated localities prove the correctness of such an opinion. Nevertheless, many towns possess advantages as residences for old people. There is more warmth in houses so situated. The congregation of so much animal life exerts a beneficial influence upon weakened and decaying constitutions. Association with younger fellow-creatures proves often advantageous to older physical frames. Besides this, aged persons, placed under the above circumstances, are more likely to receive kindness and attention from friends, and will be less likely to experience neglect, than in out of the way rural places; while the benevolent hand of charity is more freely extended. But, whatever may be the influences, very old people generally live and die in towns. To be born and brought up in the country is conducive to physical strength, and ensures ultimately a longer life, than to be born and reared in confined, densely populated, or insalubrious cities. This conclusion is based upon universal experience. In Dundee, for example, two-thirds of the individuals who became nonegenarians, are natives of rural districts, and are contradistinguished from one-third originally belonging to urban populations. One point should however be constantly kept in remembrance; viz., that if persons of very advanced years change the residences to which they have been long habituated, their career will often be materially shortened. At the same time, removal from bleak, open, and especially cold, badly sheltered country dwellings, to towns, frequently proves useful to aged constitutions.

Another observation may be based upon the data supplied, but viewed from a different aspect. If Aberdeen and Glasgow be compared, the great discrepancy which prevails respecting the ages of parties dying in each city, respectively, seems remarkable. At Glasgow, old people are comparatively rare: in Aberdeen, the contrary obtains; yet both are large manufacturing towns, situated on navigable rivers, and having each much shipping. Both are near the sea; and in many particulars they bear a marked resemblance. Aberdeen, however, lies on the east coast of Scotland; Glasgow near the western. Aberdeen possesses a dry granitic foundation, sloping towards the adjacent river; is abundantly supplied with excellent water; has a hardy, not mongrel, race of inhabitants; streets wide, well ventilated, and straight; an atmosphere little deteriorated by thick smoke, or chemical impurities; weather which, though cold and often stormy in winter, is often good at particular seasons; and lastly, but not leastly, a degree of longevity which even appears to be hereditary. Here, therefore, varied circumstances exert considerable influence on public health and human existence, tending to support the physical powers, the *vis vitæ*, and to ensure longevity. In Glasgow, on the other hand, we have exemplified the simple reverse of the above description, thus affording, *pro tanto*, an explanation of the contrast I have noticed.

My previous conclusions are all based upon mortality annals; but it would be instructive to ascertain, whether they are also supported by vital statistics. I would therefore quote the population returns of 1851, which supply important data regarding the point under discussion. In Glasgow, when the total population was stated at 329,097, there were living at that time, 81 persons above 90, making one nonagenarian in every 4,062. In Aberdeen, on the contrary, whose population amounted to 73,227 persons, 49 were 90 and upwards, which gives a ratio of one nonagenarian to every 1,494 inhabitants, or nearly three times the proportion reported to exist in Glasgow; thus proving the general truth of every former observation. In Edinburgh, which contained 59 residents aged 90 and upwards, in a population of 161,648, the ratio indicated was that of one nonagenarian in every 2,739; whilst at Perth, 10 persons, in a population of 25,441, had passed their 89th year, thus giving a proportion of one person to every 2,547 residents who had reached four score and ten, a mark of longevity superior either to Glasgow or Edinburgh, but inferior to Aberdeen. In Greenock the comparative ratio was one nonagenarian in every 7,337 inhabitants; and in Dundee, one in 4,933. Thus Aberdeen stands pre-eminent in reference to the large comparative number of very old people.

By comparing the statistics of longevity in various populous districts of England with the same statistics in Scotland, some curious facts appear. In Manchester, the proportion of people, 90 years old and upwards, during 1851, was as one to every 6,688 inhabitants; in Liverpool, one to 5,295; in Birmingham, one to 4,477; in Hull, one to 4,457; in Newcastle-upon-Tyne, one to 3,376; in London, one to 3,360; in Tynemouth, one to 1,458; and in Bath, as many as one to every 919 inhabitants, exhibiting the largest existing proportion of patriarchal residents of any locality throughout Great Britain. This peculiarity in Bath applied, however, chiefly to females: since fifty women here residing had attained, and even passed, their 90th year; whilst only nine men had reached that advanced age. But it should be recollected, that the oldest individual whose death has recently been chronicled in Scotland was a native of Bath. This was the man who died at Dundee, when 114 years old. This city must therefore be deemed salubrious, and a congenial residence for aged persons.

Instances of longevity being usually considered as more common amongst the inhabitants of Scotland, than amongst those of the southern portions of the British island, one or two remarks upon this point will not be irrelevant. Without doubt, many exceedingly old persons have been residents in north Britain; but there are no examples of such patriarchal ages as those often met with in England. The very oldest individual whose death I find recorded throughout Scotland—setting aside George Watt, previously mentioned—was a gentleman named John Rousey, who died in 1736, at Distrey, aged 138 years. The next was a gentleman named Robertson, who died near Edinburgh, in 1793, aged 137. He was an inspector of *lead* works—not the most healthy avocation —and had been so employed in these works, it is reported, during 124 years. The third Scottish patriarch was John Mount, who died at Langham, Dumfriesshire, in 1766, 136 years old. The oldest female known to have died in Scotland, was the Mary Cameron mentioned when speaking of Aberdeen; whilst the next in seniority was Margaret Scott, whose death, in 1779, at the age of 125, is recorded on her tombstone in the churchyard of Dalgeith. But instances of longevity, extending to a much longer period, have frequently been met with in England. The examples of Henry Jenkins, who died in 1670, aged 169 years: Thomas Downe, of Cheshire, in 1808, æt. 154: and Thomas Parr, æt. 152, in 1636, are well known. Besides these venerable individuals, a man named Thomas Damme, died at Leighton, near Minshul, Cheshire, in 1648, aged 154 years: another man, called James Bowls, departed this life in 1656, at the age of 152: a third, named J. Maxwell, terminated his earthly career during 1785, aged also 152 years: whilst a fourth instance of the same kind is that of a man named J. Newman, who died in 1542, at the great age of 153 years, a fact which is recorded on his tombstone in Brislington church, near Bristol. Although not altogether so old as any of the preceding instances, yet, as being the most venerable medical practitioner ever known in modern, if not ancient times, it may be well to direct attention to an altar tomb of brick, having a blue marble slab, in the grave-yard at Ware, Herts, on the south side of the church, inscribed to "William Mead, M.D., who died October 28th, 1652, aged 148 years and nine months." Dr. Mead, of Ware, may be truly considered "vetustissimus pater Britanniæ medicorum".

But, however high England may stand in reference to the very advanced ages of some of her children, the principality of Wales seems to occupy even a higher position. In Wales there are various examples of individuals who have died at ages varying from 110 to 140 years, to say nothing of the Welsh bard Llywarch Hên, the contemporary of King Arthur, who died A.D. 500, at the age of 150. A man named J. Forathe, at his death in 1621, is stated by a parish register in Glamorganshire, to have attained to 180 years. A female of the same name, viz., Elizabeth Torathe, died in 1688 after she had attained her 177th year. But the most extraordinary instance of great longevity ever recorded in Great Britain, and which must be here specially noticed, is that of an individual, respecting whose decease the parish register of Evercreech in Somersetshire contains these words: "Buried, December 20, 1588, Jane Britten, a maiden at the age of 200, as she affirmeth." If the above statement be correct, this more than ancient spinster is of historical importance, as the oldest scion of the human race ever known within any part of the British dominions.

Several foreign countries have furnished some unusual illustrations of longevity. During 1808 a negro, named J. Ram, died in Jamaica at the age of 140; a free black woman, aged also 140, died in 1812 in the same island; whilst a person designated Abraham Paiba, aged 140, died in North Carolina during the last century; besides whom another man, called J. Niblet, is reported to have died in America, in the year 1816, at the age of 143. It is further stated, on good authority, that an individual named

C. Drakenberg died in Denmark, during 1772, aged 146. A man named J. Surrington was buried in Norway, during 1797, at the age of 159; another, whose name is unknown, died in Lithuania in 1804, aged 168; whilst report says that two persons actually ended their very long lives in Russia in 1814, the one at 180, the other at the end of 200 years. But the most singular instances of great longevity occurred in the town of Temeswar, in the Banat of Hungary. In this place a peasant named Peter Torten, was laid in his grave in 1724, at the age of 185; another man called J. Rovin, died in 1741 aged 172; whilst his wife, Jane Rovin, likewise ended her days during the same year, at the great age of 164, after having been married 148 years, and having had two sons and two daughters, the youngest of whom was 116 years old.

Two other very venerable specimens of humanity are also recorded; namely, a person at first called Kentigern, according to Spotswood, but subsequently better known as St. Mongah, and after whom the famous well of St. Mungo in Wales is named, who lived till he attained his 185th year; and lastly, a man named Numas de Cugna, born in Bengal, who died in 1566, at the truly incredible age of 350 years. Maffeus, who wrote "The History of the Indies," and who has always been reckoned a model of veracity, mentions this very remarkably aged person; whilst another Portuguese author, viz., Ferdinand Lopez Casteguenda, the Historiographer Royal in Portugal, confirms this statement. The former authority mentions, among other facts, that De Cugna had four new sets of teeth, that the colour of his hair and beard had changed from black to grey, and then from grey to black again, and that the first century of his life was passed in idolatry; but having been converted to Mahometanism, he so continued until his death. If we could rely on the truth of such testimony, this more than centenarian Bengalese might be held as the true Nestor of mankind since the period of the Deluge.

Before quitting the subject of great longevity, as it has been met in modern times, I would finally remark, that although more old persons are found amongst the female sex than amongst males, at ages varying from the 60th to 110th year, yet beyond that extreme epoch, longevity is much the most common on the male side; a conclusion which may be confidently assumed from the varied facts enumerated in previous paragraphs. To prove that this inference is perfectly correct, and borne out by experience, it may be stated further, that out of 590 persons reported by various authors to have attained 110 years and upwards, I find 410 to have been men, and only 180 women; thus, giving a proportion of five males to every two females, who, having passed the unusual limit of human life just named, have become the real patriarchs of this postdiluvian world.

24, *Brook-street, Grosvenor-square,*
*December*, 1855.

